# The catastrophic financial burden of extrapulmonary tuberculosis and asset index-based inequality analysis: A prospective cohort study analysing patients’ impoverishment

**DOI:** 10.1371/journal.pgph.0005730

**Published:** 2026-02-09

**Authors:** Shoaib Hassan, Mala Kanthali, Manju Raj Purohit, Tehmina Mustafa

**Affiliations:** 1 Department of Global Public Health and Primary Care, Centre for International Health, University of Bergen, Bergen, Norway; 2 Yale School of Public Health, Yale University, New Haven, Connecticut, United States of America; 3 Department of Pathology, R.D. Gardi Medical College, Ujjain, India; 4 Department of Public Health Sciences, Karolinska Institute, Stockholm, Sweden; 5 Department of Thoracic Medicine, Haukeland University Hospital, Bergen, Norway; University of Sydney, AUSTRALIA

## Abstract

This prospective cohort study assessed the financial burden of extrapulmonary tuberculosis (EPTB) in India, revealing substantial costs across disease manifestations. Among 200 confirmed EPTB patients, those with pleuritis, meningitis, and other severe forms, incurred significantly higher out-of-pocket (OOP) and indirect costs than lymphadenitis patients. Over 90% of meningitis and 80% of pleuritis patients experienced catastrophic health expenditures (CHE), defined as costs exceeding 20% of annual income. Up to 42% of patients were pushed below the poverty line. Asset-based inequality analysis showed that lower socioeconomic groups faced a disproportionate cost burden. The cost of diagnostic tests and the less severe nature of EPTB lymphadenitis may delay seeking care until patients’ ability to work is affected. Factors such as low income, comorbidities, diagnostic delays, and reduced working capacity were associated with CHE. Notably, presumptive non-TB patients also incurred significant OOP and productivity losses during their diagnostic journeys, despite ultimately receiving non-TB diagnoses. These findings highlight the need for universal health coverage and enhanced financial protection strategies to mitigate the economic impact of EPTB and similar chronic conditions.

## Background

The current rate of decline in tuberculosis (TB) incidence is not in pace with the ambitions of the World Health Organisation (WHO) End-TB Strategy [1,2]. During 2022, about 10 million annual TB cases were reported, of which 28% were from India [3]. Although pulmonary tuberculosis (PTB) is the most common disease presentation, a large global population infected with *M. tuberculosis* also develop extrapulmonary tuberculosis (EPTB) [4]. The latest global TB report by the WHO found that EPTB cases accounted for 14–24% of notified TB cases across the WHO regions [1]. These reported cases reflect the notified case count only, and the actual EPTB cases can be as high as 40–50% among children and HIV-coinfected ones [1,5,6]. The national data from high-income countries shows that the EPTB constitutes 60% of all TB cases among people of African origin [7]. In addition to a higher proportion of EPB cases reported in the USA and Canada, there is a reported increase in EPTB cases in the European Union [8–11]. The indeterminate clinical presentation and the paucibacillary nature of EPTB add to the challenges in confirming its diagnosis, leading to a prolonged pre-diagnostic phase of illness [12–14]. Such challenges may vary by site of infection, as EPTB can present with a range of clinical manifestations, including lymphadenitis, pleuritis, meningitis, osteomyelitis, and ascites. Due to PTB’s high transmissibility potential, most research and innovation remain focused on it [1,2,15]. Furthermore, TB Control programmes mainly focus on PTB, and the substantial burden of EPTB cases is not appropriately addressed [16–18]. Therefore, EPTB cases suffer medically and economically [19,20].

Most EPTB patients in low- and middle-income settings belong to financially active younger age groups [21,22]. From an economic perspective, illnesses affecting these subgroups are particularly important and may impose financial burdens [21]. The socioeconomic implications of EPTB increase with illness duration. The cost of TB may range from 5% to 75% of patients’ annual income [23,24]. Such high costs may be defined as catastrophic health expenditures (CHE) if they exceed a certain annual income threshold, such as 20% for TB patients [25]. It is important to note that the WHO recommends estimating CHE, which is often considered an indicator of inadequate financial protection [25]. Although TB is generally known as a disease of poverty, there is limited information about how EPTB patients with various clinical manifestations bear financial hardships.

The available evidence on the financial burden of EPTB is limited, but it suggests that pre-diagnosis costs are particularly important [1,21,22,26]. A prolonged delay in diagnosing EPTB reportedly results in higher total costs for patients in both high- and low-income settings [21,22,26]. However, the available literature lacks detailed information about the components of total costs and factors associated with CHE among EPTB patients. Hence, it highlights the need to understand better the nature of the financial burden associated with EPTB. In India, the economic burden of TB-related illness could be high, given that it is one of the high TB burden countries. Given the lack of general information on patient-incurred costs of EPTB illness in high-burden settings, we planned this study. The main aim was to study how presumptive EPTB patients experience financial impacts during the pre-diagnostic and treatment phases of illness, and to assess factors associated with the CHE and the inequity in incurred costs among patients with various socioeconomic positions (SEPs) categorised by clinical manifestations.

## Methods

### Study setting and data collection

This cohort study was part of a larger project at the 750-bed Chandrikaben Rashimikant Gardi Hospital (CRGH), which is affiliated with Ruxmaniben Deepchand Gardi Medical College (RDGMC) in Ujjain, Madhya Pradesh, India. From November 2018 to February 2020, patients of all ages with presumptive extra-pulmonary tuberculosis (EPTB) were prospectively enrolled in the study. Individuals who did not provide informed written consent or had undergone anti-tuberculosis treatment (ATT) in the past year were excluded.

Under a specialist doctor’s guidance, two trained hospital staff members interviewed the participants using a pre-designed questionnaire. Patients could respond in Hindi or local dialects. Adults provided their responses, while caregivers answered on behalf of children. Privacy was ensured throughout the interview process. Experts reviewed the questionnaire, while the interviewing staff gathered demographic, socioeconomic, and clinical data. Additionally, it included questions about income loss due to EPTB, productivity loss, and expenses incurred by patients and their accompanying family members.

After enrolment and the interview, qualified doctors conducted relevant clinical examinations and recorded their observations. All presumptive EPTB patients underwent diagnostic testing, with their diagnoses confirmed using a validated composite reference standard (CRS) [27]. Patients with confirmed EPTB were treated according to the guidelines set by the Indian National Tuberculosis Control Programme [28]. After treatment was initiated, EPTB patients had follow-up clinical visits to monitor disease progression and treatment effectiveness. The follow-up period lasted up to ten months for meningitis patients and six months for those with lymphadenitis, pleuritis, ascites, or osteomyelitis. Patients who did not begin anti-tuberculosis treatment (ATT) were monitored until they either recovered or received a diagnosis unrelated to TB.

All collected data was transferred from hard copies to an electronic format using Microsoft Excel. To ensure accuracy, each patient was assigned a unique identifier to prevent duplication, and two researchers randomly cross-checked 10% of the data entries for internal validation.

### Study participants and EPTP-related cost estimation

Our study participants included EPTB and non-TB patients, categorised according to the CRS. We performed a descriptive analysis of demographic, socioeconomic, and clinical information to identify significant differences (p-value < 0.05, Chi-square test) between EPTB and non-TB patients. We checked for normal distribution using the Shapiro-Wilk test. Then, we applied the Kruskal-Wallis test to assess significant differences (p-value < 0.05) in the medians of non-normally distributed data. The key terminology used in this study is below.

- Pre-diagnostic phase: The time between a patient’s first notice of EPTB signs or symptoms and the diagnostic confirmation.
- Treatment phase: The period from ETPB diagnostic confirmation to the end of TB treatment.
- Diagnostic site: Our study site, where presumptive EPTB patients received diagnostic confirmation (positive or negative).
- Diagnostic visit: One of the visits to the diagnostic site when the presumptive EPTB patients received their diagnostic confirmation.
- Follow-up visits: During the treatment phase, EPTB patients made follow-up visits to the diagnostic site for clinical evaluation and to collect TB medications.

From a patient perspective, we calculated costs incurred by all the presumptive EPTB patients during the pre-diagnostic phase of illness. After the diagnosis, appropriate TB treatment was provided free of cost to EPTB patients as per the TB control program guidelines [28]. Therefore, the cost estimates during the treatment phase only comprised transport costs for EPTB patients’ follow-up visits. We did not systematically follow up with non-TB patients for their treatment, whose costs incurred during the treatment phase are not included in this study. This study adopted a methodology of direct and indirect cost estimation as follows [25].

- The direct costs during the pre-diagnostic phase covered both medical (consultation, admission, diagnostic tests, and any non-TB medication for the ongoing illness) and non-medical (patient transportation costs) out-of-pocket (OOP) expenditures. The direct cost during the treatment phase for EPTB patients included transportation for follow-up visits. Based on the derived cost of the diagnostic visit, we extrapolated transportation costs for the minimum recommended follow-up visits per the National Tuberculosis Programme in India for various EPTB manifestations: three for lymphadenitis, pleuritis, abdominal and five for meningitis TB [28]. Although some patients may have additional visits, we opted for these conservative estimates. We did not have information on the costs of food items and the caregiver’s transportation to HF visits, which were not included in the total direct cost.
- The indirect (opportunity) cost estimation during the pre-diagnostic phase was based on the most common method for this purpose, the input-based Human Capital Approach. This method valued forgone productivity based on the reported average income before the EPTB illness [25]. These cost estimates consisted of lost income due to the forgone productivity of both the lost working capacity of EPTB patients and their caregivers (when involved) during the pre-diagnostic phase. To estimate indirect costs, we used information reported during the interviews about the duration (days) and extent (percentage) of lost working capacity of patients and their caregivers. We derived daily income from the patient-reported average annual income of their self-reported salary category during the previous year, assuming 22 working days per month and eight hours per day. For children under 15 years old and caregivers of adult patients, we used the same daily income as their parents’ and patients’ income, respectively. The indirect income resulted from the duration of lost working capacity multiplied by the percentage of lost working capacity and daily income. During the treatment phase, we did not have information on the lost work-days productivity of EPTB patients or their caregivers, which was not included in the indirect cost estimates.
- In addition to the cost of follow-up visits, we estimated the cost of lost productivity (time lost from work due to travel and waiting) during the diagnostic visit. To calculate this cost, we multiplied the time spent travelling to and waiting (hours) at the diagnostic facility by the hourly income derived from the estimated monthly income. This cost estimation was included in the indirect cost.

The total costs included direct and indirect costs mentioned during the pre-diagnostic and treatment phases.

- **Direct costs** included the following:
  - Medical expenses (consultation, admission, diagnostic tests, and non-TB medication)
  - Non-medical expenses (such as patient transportation) during the pre-diagnostic phase.
  - Transportation for recommended follow-up visits during the treatment phase.
  • Costs for food and caregiver transportation were not included due to the unavailability of information.
- **Indirect costs** included the following:
  - Lost productivity of patients and caregivers during the pre-diagnostic phase.
    - ◦ This was calculated based on reported income, duration, and percentage of lost working capacity. Productivity loss during the treatment phase was not included in the estimates due to the unavailability of information.

We compared the costs mentioned above between EPTB and non-TB patients during the pre-diagnostic phase of illness. Then, we analysed median costs with interquartile ranges (IQRs) across EPTB patient subgroups categorised by disease manifestations. Moreover, we estimated the proportions of contributors to the direct, indirect, and total costs to assess significant differences (p-value < 0.05, Kruskal-Wallis test). We collected all costs in Indian Rupees (INR) and converted them to USD using the 2024 exchange rate of USD 1 = 83.7 INR [29]. We also standardised the cost inputs to a single base year of 2023 using the latest World Bank annual GDP deflator for India [30,31].

### Catastrophic health expenditures

We calculated the proportion of patients encountering CHE. The WHO-recommended threshold for estimating catastrophic costs due to TB is when total costs exceed 20% of the average household’s annual income [25]. We recorded the patient’s self-reported annual income (instead of household income). We used it to estimate the base-case CHE as the proportion of total costs above 20% of the patient’s average annual income before EPTB diagnosis. Then, we conducted a sensitivity analysis and estimated CHE across various thresholds to account for potential variation in patient and household income. This included calculating the proportion of patients who incurred total costs equal to or exceeding 10% and 50% of their average annual income. Moreover, we estimated the proportion of patients whose OOP expenditures (direct costs) exceeded 10% and 20% of their average annual income. In this study, we did not record information on coping mechanisms to offset costs; therefore, this cost estimate did not include them.

### Risk factors analysis for catastrophic health expenditures

We used univariable logistic regression to examine demographic, socioeconomic, and clinical factors potentially associated with the base-case CHE. Factors with a significant association (Odds Ratio (OR), p < 0.05) in the univariable regression analysis were included in the multivariable regression model. We reported the final model with a statistically significant (p-value < 0.05) adjusted odds ratio (aOR) and 95% confidence intervals (CI). We conducted all statistical analyses using R software employing the ggplot, tidyverse and LorenzRegression packages [32–34].

### Socioeconomic positions: Based on the asset index

As stated earlier, we recorded self-reported categories of patients’ salaries rather than annual income as a continuous variable. Therefore, to obtain a variable that could reflect long-term SEPs, we conducted a principal component analysis (PCA) as a data reduction method to calculate an asset index based on household ownership [35]. The asset index considered a range of variables: the number of family members living, owning a house, source of drinking water, type of toilet facility, electricity, gas, radio, television, telephone/mobile, iron, refrigerator, source of energy for lighting, mode of transportation, bank account, land for farming as well as material of walls and roof of the house. Traditionally, the PCA allows the extraction of uncorrelated variables as principal components (PC) scores [36]. Each component describes variation in the dataset in order such that the first PC (PC1) explains the most variability in household ownership data [37]. The following components explain the additional variation, but to a lesser extent than the first component. We utilised the PC1 score (also called a dimension), a proxy indicator of the asset index as per the established PCA methodology [35,38].

### Inequality analysis

The concentration index (CI) has an established methodology for assessing inequalities in health within a population ranked by their SEPs [39–41]. Similarly, we estimated financial inequalities among sub-groups of EPTB patients categorised by their clinical manifestation. The CI is twice the area between the line of equality (the 45^o^ line and the concentration curve (CC)). The value of CI ranges from -1–1. Conventionally, the index takes negative and positive values when the CC lies above and below the line of equality, respectively. This means a negative index indicates a higher concentration of the outcome among the poor, while a positive index indicates a higher concentration among the richer SEPs. The 45^o^ line would have a zero value of CI. Formally, the CI is defined as


$$C=\frac{\text{2}}{\mu }+\text{cov}(y\;,\;R)$$

The μ and cov referred to the means and covariances of the outcome variables belonging to the study population. The y is an individual’s outcome (financial burden in our study), while *R* is the individual’s rank per the SEP.

### EPTB-related costs and the poverty line

We estimated the proportion of EPTB patients who fell below the poverty line based on their remaining income after the EPTB expenditures. For this purpose, we subtracted EPTB-related total costs from the average annual salary and calculated the proportion of patients whose remaining income was below the poverty line. If we used the World Bank-reported international poverty line of USD 2.15 per person per day income as the threshold for this analysis [42], all patients except the highest category would be below the poverty line even before accounting for any EPTB-related expenditures. Therefore, in line with the recent literature, we used India’s national poverty line, which is better suited to our data from the rural settings [43–46]. The latest available poverty line thresholds, as per the Rangarajan committee, are INR 972 (USD 12) and INR 1407 (USD 17) per person per month in rural and urban settings, respectively [45,47]. We converted INR 972 to USD (the 2024 exchange rate of USD 1 = INR 83.7) and standardised it to a single base year of 2023 per the latest World Bank annual GDP deflator for India [29–31]. Finally, our poverty line was set at INR 1326 per person per month (USD 164 annually).

### Ethics

We obtained informed consent from all adult patients and parents or guardians of minors. As part of the clinical workup, before testing for HIV, both verbal and written consent were obtained. The study was approved by the Regional Committee for Medical Research Ethics in Norway (Ref: 2014/46/REK vest) and the ethical committee in India (Ref: IEC 10/2018). Patients or the public were not involved in the design, reporting, or dissemination plans for our research.

## Results

### Sociodemographic and clinical characteristics of EPTB vs non-TB patients

Our results are based on questionnaire data from 330 presumptive EPTB patients. Among these, 200 were categorised as EPTB and 130 non-TB patients according to the CRS (Table 1). Compared to non-TB patients, the EPTB patients were significantly different (p-value <0.05), including a higher proportion of females (61%), 16–45 years old adults (73%) and pleuritis manifestations (23%). Similarly, a relatively higher number of EPTB patients reported a family history of TB (19%), the total number of visits to HFs (1–3 among 75% of patients), the longer time taken to reach the nearest HF (>30 minutes among 63%), and lost capacity to work (>25% among 89% patients) due to the ongoing illness (Table 1). We categorised patients into six income groups (labelled very low, low, low-middle, high-middle, high and very high) corresponding to the average annual income of their self-reported income categories: INR 5000, 15000, 25000, 35000, 45000 and 55000.

**Table 1:** Demographic, socioeconomic and clinical characteristics of presumptive EPTB patients.

		EPTB patients (%)	Non-TB patients (%)	p-value*
Total N (%)		200 (60.6)	130 (39.4)	
Disease manifestations	Lymphadenitis	142 (71.0)	107 (82.3)	0.002
	Pleuritis	40 (20.0)	10 (7.7)	
	Meningitis	12 (6.0)	13 (10.0)	
	Others	6 (3.0)	0 (0.0)	
Gender	Female	121 (60.5)	59 (45.4)	0.010
	Male	79 (39.5)	71 (54.6)	
Age groups	Under 15 yr	22 (11.0)	34 (26.2)	<0.001
	16-29 yr	103 (51.5)	38 (29.2)	
	30-44 yr	42 (21.0)	26 (20.0)	
	Above 45 yr	33 (16.5)	32 (24.6)	
Education levels	Primary or below	121 (60.5)	88 (67.7)	0.394
	Middle or Secondary	56 (28.0)	31 (23.8)	
	Higher	23 (11.5)	11 (8.5)	
Marital status	Married	119 (66.9)	66 (66.0)	0.990
	Unmarried	59 (33.1)	34 (34.0)	
Household family members	1-4	68 (34.0)	37 (28.5)	0.393
	5-7	97 (48.5)	73 (56.2)	
	>=8	35 (17.5)	20 (15.4)	
Salary categories	Very low	9 (4.5)	9 (6.9)	0.392
	Low	17 (8.5)	11 (8.5)	
	Low-middle	15 (7.5)	14 (10.8)	
	High-middle	37 (18.5)	26 (20.0)	
	High	69 (34.5)	31 (23.8)	
	Very high	53 (26.5)	39 (30.0)	
Occupation	Govt Employed	36 (18.0)	33 (25.4)	0.192
	Private Employed	53 (26.5)	32 (24.6)	
	Housewife	64 (32.0)	30 (23.1)	
	Unemployed	47 (23.5)	35 (26.9)	
Previous history of TB	No	176 (88.0)	122 (93.8)	0.118
	Yes	24 (12.0)	8 (6.2)	
Family history of TB	No	163 (81.5)	120 (92.3)	0.010
	Yes	37 (18.5)	10 (7.7)	
Self-medication for this illness	No	160 (80.0)	110 (84.6)	0.360
	Yes	40 (20.0)	20 (15.4)	
Hospitalisation status for this illness	Inpatient	79 (39.5)	42 (32.3)	0.227
	Outpatient	121 (60.5)	88 (67.7)	
Co-morbidities (DM, HIV, history of TB)	No	161 (80.5)	114 (87.7)	0.118
	Yes	39 (19.5)	16 (12.3)	
Total delay in diagnosis	Under 1 week	25 (12.5)	17 (13.1)	0.105
	2-4 weeks	91 (45.5)	75 (57.7)	
	1-6 months	76 (38.0)	33 (25.4)	
	Above 6 months	8 (4.0)	5 (3.8)	
Number of HFs previously visited for this illness	One	69 (34.5)	30 (23.1)	0.034
	Two-three	81 (40.5)	54 (41.5)	
	Four	0 (0.0)	2 (1.5)	
	Missing	50 (25.0)	44 (33.8)	
Number of previous visits to HF for this illness	One	59 (29.5)	28 (21.5)	0.182
	Two-Four	99 (49.5)	66 (50.8)	
	Missing	42 (21.0)	36 (27.7)	
Time to reach the nearest HF	Below 30 min	74 (37.0)	66 (50.8)	0.015
	Between 30–60 min	76 (38.0)	46 (35.4)	
	Above 60 min	50 (25.0)	18 (13.8)	
Time to reach the diagnostic site	Below 30 min	96 (48.0)	74 (56.9)	0.158
	30- 60 min	56 (28.0)	25 (19.2)	
	Above 60 min	48 (24.0)	31 (23.8)	
Total time (travel & wait) at the diagnostic site	Below 60 min	115 (57.5)	86 (66.2)	0.145
	Above 60 min	85 (42.5)	44 (33.8)	
HFs previously visited for this illness	Dispensary	98 (49.0)	76 (58.5)	0.130
	Health Center/District/Others	23 (11.5)	8 (6.2)	
	Regional/Private Hospital	79 (39.5)	46 (35.4)	
Reduction in working capacity duration (days)	Below 15 days	92 (46.7)	61 (48.8)	0.800
	Above 15 days	105 (53.3)	64 (51.2)	
Reduction in working capacity extent (%)	Below 25%	22 (11.1)	45 (35.7)	<0.001
	25- 50%	95 (48.0)	58 (46.0)	
	Above 50%	81 (40.9)	23 (18.3)	

* p-value corresponds to the Kruskal-Wallis test.

### EPTB patients by disease manifestations

The EPTB patients reported several disease manifestations: lymphadenitis (71%), pleuritis (20%), meningitis (6%), and others (3% comprising ascites (n = 4) and osteomyelitis (n = 2)). Based on the descriptive analysis categorised by these manifestations, EPTB patients differed significantly from non-TB patients across various factors (p-value < 0.05), as detailed in Table 2. More females (68%) presented with lymphadenitis than males with pleuritis (56%) or meningitis (75%). Young adult (16–29 years old) patients mainly presented with lymphadenitis (56%) and pleuritis (50%), whereas patients aged>45 years had a higher proportion with meningitis (42%) and others (83%). A higher proportion of patients with all disease manifestations had a primary education level or below (54%-100%). In terms of healthcare access, most patients had 1–2 visits to HFs, except 15% of pleuritis patients who had up to three trips to HFs for this illness. Patients with lymphadenitis and pleuritis reported a longer time (>30 minutes) to reach the nearest HF and the diagnostic site. A shorter combined journey and wait time (<10 minutes) to the diagnostic site was reported mainly among patients with lymphadenitis. Patients with pleuritis and meningitis had higher inpatient hospitalisation rates (88% and 92%, respectively) than those with lymphadenitis (20%). The patients’ reported extent (%) of EPTB illness, in terms of reduced working capacity, was above 25% in all except for a smaller proportion of patients with lymphadenitis (15%) and pleuritis (3%) (Table 2).

**Table 2:** Main characteristics of EPTB patients as categorised by their disease manifestations.

		Lymphadenitis	Pleuritis	Meningitis	Others*	p-value**
Total N (%)		142 (71.0)	40 (20.0)	12 (6.0)	6 (3.0)	
Gender	Female	96 (67.6)	17 (42.5)	3 (25.0)	5 (83.3)	0.001
	Male	46 (32.4)	23 (57.5)	9 (75.0)	1 (16.7)	
Age groups	Under 15 yr	20 (14.1)	1 (2.5)	1 (8.3)	0 (0.0)	<0.001
	16-29 yr	79 (55.6)	20 (50.0)	3 (25.0)	1 (16.7)	
	30-44 yr	28 (19.7)	11 (27.5)	3 (25.0)	0 (0.0)	
	Above 45 yr	15 (10.6)	8 (20.0)	5 (41.7)	5 (83.3)	
Education level	Primary or below	76 (53.5)	29 (72.5)	10 (83.3)	6 (100.0)	0.026
	Middle or Secondary	44 (31.0)	10 (25.0)	2 (16.7)	0 (0.0)	
	Higher	22 (15.5)	1 (2.5)	0 (0.0)	0 (0.0)	
Marital status	Married	77 (62.6)	29 (74.4)	7 (70.0)	6 (100.0)	0.171
	Unmarried	46 (37.4)	10 (25.6)	3 (30.0)	0 (0.0)	
Household family members	1-4	49 (34.5)	13 (32.5)	5 (41.7)	1 (16.7)	0.553
	5-7	72 (50.7)	16 (40.0)	5 (41.7)	4 (66.7)	
	>=8	21 (14.8)	11 (27.5)	2 (16.7)	1 (16.7)	
Salary categories	Very low	8 (5.6)	0 (0.0)	1 (8.3)	0 (0.0)	0.304
	Low	13 (9.2)	3 (7.5)	1 (8.3)	0 (0.0)	
	Low-middle	8 (5.6)	5 (12.5)	1 (8.3)	1 (16.7)	
	High-middle	23 (16.2)	12 (30.0)	2 (16.7)	0 (0.0)	
	High	55 (38.7)	7 (17.5)	5 (41.7)	2 (33.3)	
	Very high	35 (24.6)	13 (32.5)	2 (16.7)	3 (50.0)	
Occupation	Govt Employed	24 (16.9)	8 (20.0)	2 (16.7)	2 (33.3)	0.167
	Private Employed	31 (21.8)	15 (37.5)	6 (50.0)	1 (16.7)	
	Housewife	50 (35.2)	10 (25.0)	1 (8.3)	3 (50.0)	
	Unemployed	37 (26.1)	7 (17.5)	3 (25.0)	0 (0.0)	
Patient's history of TB	No	121 (85.2)	38 (95.0)	11 (91.7)	6 (100.0)	0.276
	Yes	21 (14.8)	2 (5.0)	1 (8.3)	0 (0.0)	
Family member’s history of TB	No	116 (81.7)	32 (80.0)	10 (83.3)	5 (83.3)	0.991
	Yes	26 (18.3)	8 (20.0)	2 (16.7)	1 (16.7)	
Self-medication	No	115 (81.0)	32 (80.0)	8 (66.7)	5 (83.3)	0.691
	Yes	27 (19.0)	8 (20.0)	4 (33.3)	1 (16.7)	
Hospitalisation status	Inpatient	28 (19.7)	35 (87.5)	11 (91.7)	5 (83.3)	<0.001
	Outpatient	114 (80.3)	5 (12.5)	1 (8.3)	1 (16.7)	
Co-morbidities (DM, HIV, history of TB)	No	108 (76.1)	32 (80.0)	10 (83.3)	4 (66.7)	0.823
	Yes	34 (23.9)	8 (20.0)	2 (16.7)	2 (33.3)	
Total delay in diagnosis	Under 1 week	15 (10.6)	5 (12.5)	3 (25.0)	2 (33.3)	0.604
	2-4 weeks	66 (46.5)	20 (50.0)	4 (33.3)	1 (16.7)	
	1-6 months	54 (38.0)	14 (35.0)	5 (41.7)	3 (50.0)	
	Above 6 months	7 (4.9)	1 (2.5)	0 (0.0)	0 (0.0)	
Number of HFs previously visited for this illness	One	50 (35.2)	13 (32.5)	2 (16.7)	4 (66.7)	0.384
	Two	52 (36.6)	12 (30.0)	6 (50.0)	1 (16.7)	
	Three	5 (3.5)	3 (7.5)	1 (8.3)	1 (16.7)	
	Four	0 (0.0)	0 (0.0)	0 (0.0)	0 (0.0)	
	Missing	35 (24.6)	12 (30.0)	3 (25.0)	0 (0.0)	
Number of previous visits to HFs for this illness	One	46 (32.4)	10 (25.0)	0 (0.0)	3 (50.0)	0.035
	Two	58 (40.8)	12 (30.0)	7 (58.3)	2 (33.3)	
	Three	8 (5.6)	6 (15.0)	1 (8.3)	0 (0.0)	
	Four	1 (0.7)	2 (5.0)	1 (8.3)	1 (16.7)	
	Missing	29 (20.4)	10 (25.0)	3 (25.0)	0 (0.0)	
Time to reach the nearest HF	Below 30 min	57 (40.1)	12 (30.0)	3 (25.0)	2 (33.3)	0.001
	Between 30–60 min	62 (43.7)	8 (20.0)	5 (41.7)	1 (16.7)	
	Above 60 min	23 (16.2)	20 (50.0)	4 (33.3)	3 (50.0)	
Time to reach the diagnostic site	Below 30 min	74 (52.1)	16 (40.0)	3 (25.0)	3 (50.0)	0.035
	30- 60 min	43 (30.3)	9 (22.5)	4 (33.3)	0 (0.0)	
	Above 60 min	25 (17.6)	15 (37.5)	5 (41.7)	3 (50.0)	
Total time (travel & wait) at the diagnostic site	Below 10 min	110 (77.5)	4 (10.0)	0 (0.0)	1 (16.7)	<0.001
	10- 60 min	23 (16.2)	10 (25.0)	4 (33.3)	1 (16.7)	
	60- 120 min	6 (4.2)	19 (47.5)	7 (58.3)	1 (16.7)	
	Above 120 min	3 (2.1)	7 (17.5)	1 (8.3)	3 (50.0)	
HFs previously visited for this illness	Dispensary	72 (50.7)	19 (47.5)	5 (41.7)	2 (33.3)	0.696
	District Hospital	12 (8.5)	3 (7.5)	2 (16.7)	1 (16.7)	
	Health Center/Others	4 (2.8)	1 (2.5)	0 (0.0)	0 (0.0)	
	Private Hospital	4 (2.8)	0 (0.0)	0 (0.0)	1 (16.7)	
	Regional Hospital	50 (35.2)	17 (42.5)	5 (41.7)	2 (33.3)	
Reduction is working capacity duration (days)	Below 15 days	65 (46.8)	20 (50.0)	4 (33.3)	3 (50.0)	0.227
	16- 30 days	42 (30.2)	13 (32.5)	8 (66.7)	2 (33.3)	
	Above 30 days	32 (23.0)	7 (17.5)	0 (0.0)	1 (16.7)	
Reduction is working capacity extent (%)	Below 25%	21 (15.0)	1 (2.5)	0 (0.0)	0 (0.0)	<0.001
	25- 50%	84 (60.0)	8 (20.0)	2 (16.7)	1 (16.7)	
	51- 75%	27 (19.3)	14 (35.0)	2 (16.7)	1 (16.7)	
	76- 100%	8 (5.7)	17 (42.5)	8 (66.7)	4 (66.7)	

*Ascites (4) & Osteomyelitis (2) patients, ** p-value corresponds to chi-square test.

During the pre-diagnostic phase of illness, the patient-reported median direct costs (OOP) incurred by the EPTB patients were as follows: admission USD 0 (IQR 0.0-5.1), consultation USD 3.3 (IQR 0.0-7.2), non-TB medication USD 7.2 (IQR 3.1-20.6), diagnostics USD 4.1 (IQR 1.5-10.3) and transportation USD 2.1 (IQR 0.5-3.3). For the non-TB patients, the median costs were as follows: admission USD 0.0 (IQR 0.0-0.0), consultation USD 1.0 (IQR 0.0-5.1), non-TB medication USD 5.1 (IQR 2.1-10.3), diagnostics USD 2.8 (IQR 1.3-6.2) and transportation USD 1.0 (IQR 0.5-2.1). The only significant difference in costs between EPTB and non-TB patients was the consultation cost (p < 0.005).

### Estimation of EPTB-related costs

**Fig 1:**
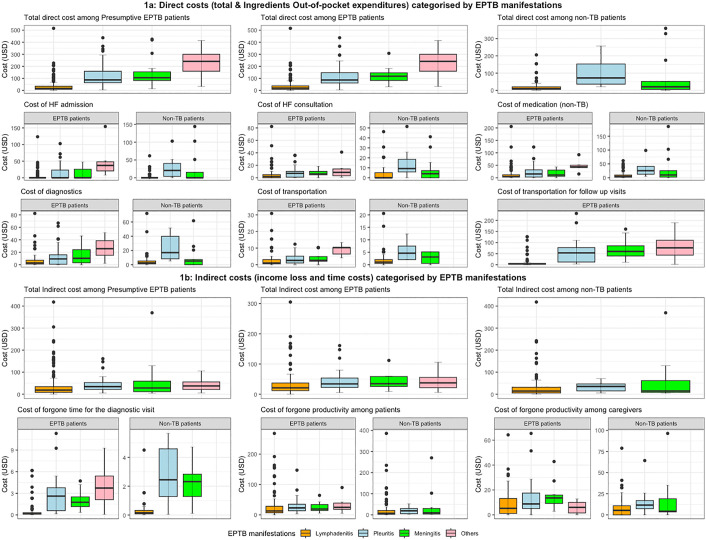
(A) Direct costs (out-of-pocket expenditures) incurred by presumptive EPTB patients, categorised by the disease manifestations*. *(Cost of admission: costs incurred for the HF admission, Cost of medication: cost of non-TB medication that patients received as a treatment before being correctly diagnosed as TB patient, Cost of diagnostics: cost of laboratory tests to diagnose the ongoing illness, Cost of transportation: cost of transportation to visit HFs before the TB diagnosis, Cost of transportation for follow up visits: cost of transportation during the treatment phase for follow-up visits). (B) Indirect costs incurred by EPTB patients, categorised by the disease manifestations.

Analysing these pre-diagnostic phase costs by disease manifestation showed that among EPTB patients, the median costs were highest among those labelled as others (ascites and osteomyelitis). Otherwise, the EPTB and non-TB patients followed a similar pattern: highest for pleuritis, followed by meningitis and lymphadenitis (Fig 1A). During the pre-diagnostic phase, the highest cost incurred by EPTB patients was due to non-TB medication. The second-highest cost among all disease manifestations was for diagnostics, except for patients in the other category, whose hospital admission costs were higher than those for diagnostics. Otherwise, during the pre-diagnostic phase, the cost of clinical consultation was the third highest, followed by transportation costs and HF admission (Fig 1A). During the treatment phase, the median transportation cost for follow-up visits among all EPTB patients was estimated at USD 5.6 (IQR 2.6-24.5). By disease manifestation, this cost component was highest among patients categorised as others, followed by meningitis, pleuritis and lymphadenitis (Fig 1A).

The median time for diagnostic visits among EPTB and non-TB patients with various disease manifestations was 6–108 and 6–84 hours, respectively (Table A in S1 Text). The median indirect costs for both EPTB and non-TB patients were highest for forgone productivity (USD 16.1 & USD 7.9), followed by family members as caregivers (USD 7.4 & USD 5.6) and the diagnostic visit (USD 0.3 & USD 0.2). Fig 1B shares more detailed information on indirect costs by disease manifestations. Among the EPTB patients, the median total direct cost (pre-diagnostic and treatment phase OOP expenditures) was significantly different among patients of various disease manifestations (p < 0.001, Table B in S1 Text): meningitis USD 118 (IQR 82–146), followed by pleuritis USD 86 (IQR 61–146) and lymphadenitis USD 19 (IQR 8–37).

The median total indirect cost varied significantly by EPTB manifestation: meningitis USD 35 (IQR 26–59); pleuritis USD 34 (IQR 23–54); and lymphadenitis USD 21 (IQR 12–37). The median direct and indirect costs for other patients (four with ascites and two with osteomyelitis) were USD 241 (IQR 159–300) and USD 38 (IQR 22–56), respectively. Analysing the proportion of various contributors of the total direct costs incurred by the EPTB patients due to their ongoing illness were significantly highest for the follow-up visits during the treatment phase (29%), followed by pre-diagnostic phase non-TB medication (17%), diagnostics (11%) and consultations (10%) and admission (9%) as elaborated in the Table C in S1 Text.

### Catastrophic health expenditures

The base-case analysis of CHE among EPTB patients (>20% of average annual income spent on total EPTB costs) showed a high proportion of meningitis (92%), pleuritis (80%) and others (83%) compared with lymphadenitis (26%) patients who incurred CHE. A sensitivity analysis of the similar CHE at 10% and 50% of annual income also showed significant CHE patterns (p-value <0.05). Additional information about these analyses is presented in Table 3. Moreover, we estimated CHE as direct costs (OOP expenditures) using thresholds of 10% and 20% of average annual income. At the 10% threshold, a higher proportion of meningitis and pleuritis patients (>50%) and others (100%) incurred CHE (Table 3).

**Table 3:** EPTB patients’ catastrophic health expenditures by disease manifestations.

		Lymphadenitis	Pleuritis	Meningitis	Others	*p-value
Total cost >20% of annual income	Catastrophic costs	36 (26.1)	32 (80.0)	11 (91.7)	5 (83.3)	<0.001
	No Catastrophic costs	102 (73.9)	8 (20.0)	1 (8.3)	1 (16.7)	
Total cost >10% of annual income	Catastrophic costs	81 (58.7)	35 (87.5)	12 (100.0)	6 (100.0)	<0.001
	No Catastrophic costs	57 (41.3)	5 (12.5)			
Total cost >50% of annual income	Catastrophic costs	9 (6.5)	13 (32.5)	6 (50.0)	4 (66.7)	<0.001
	No Catastrophic costs	129 (93.5)	27 (67.5)	6 (50.0)	2 (33.3)	
Direct cost (OOP) >10% of annual income	Catastrophic costs	28 (19.7)	20 (50.0)	7 (58.3)	6 (100.0)	<0.001
	No Catastrophic costs	114 (80.3)	20 (50.0)	5 (41.7)		
Direct cost (OOP) >20% of annual income	Catastrophic costs	11 (7.7)	15 (37.5)	5 (41.7)	4 (66.7)	<0.001
	No Catastrophic costs	131 (92.3)	25 (62.5)	7 (58.3)	2 (33.3)	

* p-value corresponds to the chi-square test.

### Factors associated with the catastrophic health expenditures

**Fig 2:**
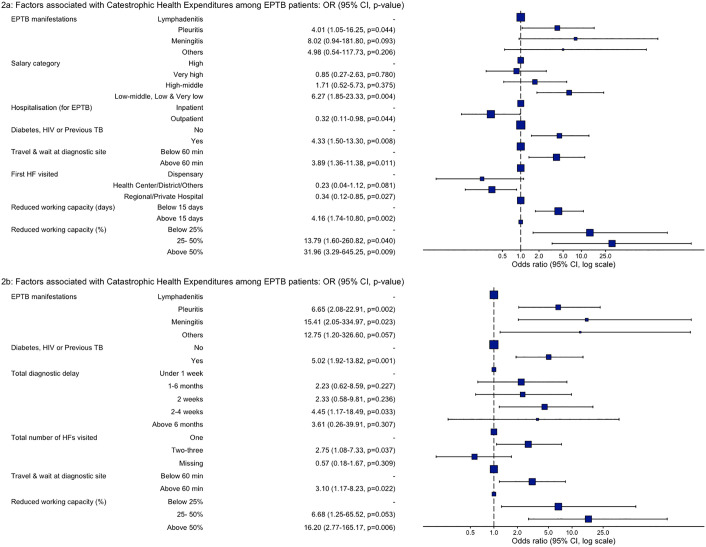
A-B Multivariable regression models presenting factors associated with the base-case CHE.

We conducted the regression analysis to identify factors associated with the base-case CHE (Table D in S1 Text: Number in dataframe = 196, Number in model = 177, Missing = 19, Akaike Information Criterion (AIC) = 187.6). The final model presented factors significantly associated with the CHE (Fig 2A): Pleuritis (aOR 4.01, 95% CI 1.05-16.25) compared to lymphadenitis, low-middle to very low salaried (aOR 6.27, 95% CI 1.85-23.33) compared to the high salary group, comorbidities such as Diabetes, HIV or previous TB (aOR 4.33, 95% CI 1.50-13.30) compared to no such illnesses and travel & wait time at the diagnostic site of >60 minutes (aOR 3.89, 95% CI 1.36-11.38) compared to below 60 minutes. Similarly, another factor associated with the CHE was an EPTB-related reduction in working capacity lasting more than 15 days (aOR 4.16, 95% CI 1.74-10.80) compared to less than 15 days. Moreover, reductions in working capacity of 25–50% (aOR 13.79, 95% CI 1.60-260.82) and >50% (aOR 31.96, 95% CI 3.29-645.25) compared to below 25% were also associated with the CHE. Our final model also included factors protecting from the CHE: outpatients (aOR 0.32, 95% CI 0.11-0.98) compared to inpatients and going to the regional hospital or a private HF (aOR 0.34, 95% CI 0.12-0.85) compared to dispensaries as the first HF attended for the ongoing EPTB illness.

We also ran the regression analysis without variables that could be correlated with the CHE. For this purpose, we excluded the patient’s salary and the duration of lost working capacity from the second regression model. However, the results remained the same as those of the first regression model (Fig 2B). However, the second model indicated that, compared to EPTB diagnosis within a week, a total diagnostic delay of 2–4 weeks was significantly associated with the CHE (aOR 4.45, 95% CI 1.17-18.49). Similarly, this model indicated that compared to visiting a single HF for EPTB illness, patients visiting 3–4 HFs were associated with the CHE (aOR 2.75, 95% CI 1.08-7.33).

### Asset index-based inequality in EPTB-related costs

**Fig 3:**
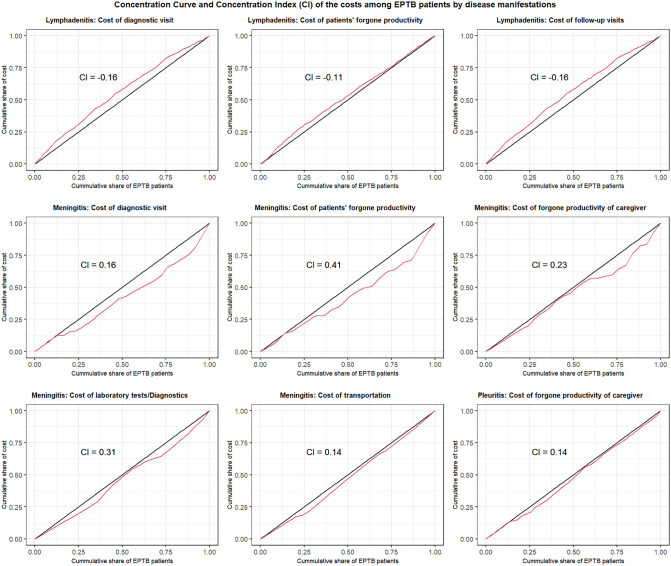
Distribution of costs by EPTB manifestations and asset index (A curve above the line of equality indicates concentration among patients of lower SEP, while a curve below it indicates concentration among patients of higher SEP).

We estimated the asset index based on the PC1 score employing the PCA of household ownership. Then, we used the asset index for the inequality analysis per the CC and the CI. We found inequality in the distribution of a few contributors of the direct and indirect costs among EPTB patients categorised by disease manifestation (Fig 3). For example, we found a large cumulative proportion of lymphadenitis EPTB patients from the lower SEP facing a higher concentration of costs of (i) diagnostic visits, (ii) illness-related forgone productivity of patients and (iii) follow-up visits (Fig 3). On the other hand, several of the costs were concentrated among meningitis EPTB patients of higher SEP (Fig 3), such as costs of (i) diagnostic visits, (ii) illness-related forgone productivity of patients, (iii) forgone productivity of a family member caregiver among the patients of high SEP, (iv) laboratory tests/diagnostics and (v) transportation. The EPTB pleuritis patients with high SEP had a higher concentration of the cost of forgone productivity of a family member caregiver. Fig 3 presents costs concentrated in either the low or high SEPs.

### EPTB-related costs leading to impoverishment: patients falling below the poverty line

**Fig 4:**
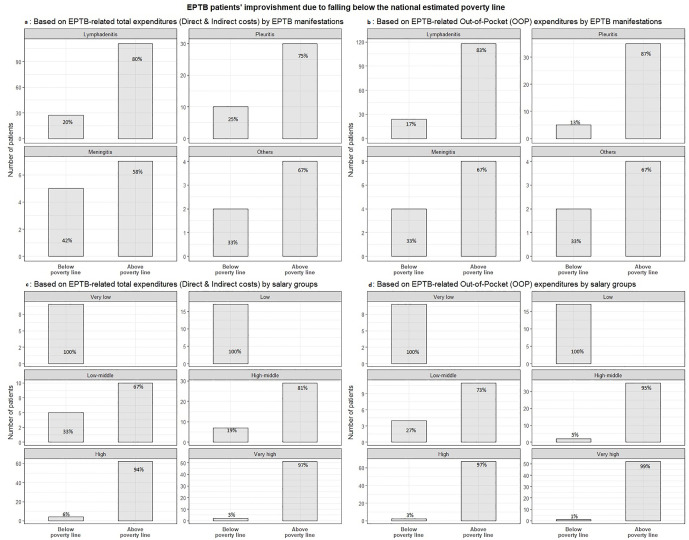
EPTB patients above and below the poverty line based on total costs and Out-of-pocket expenditures.

Based on the methodology mentioned above, accounting for patients’ average annual income and the EPTB-related total expenditure incurred, our analysis showed that EPTB patients of all manifestations were vulnerable to impoverishment and fell below the poverty line: lymphadenitis (20%), pleuritis (25%), meningitis (42%), and others (33%) as shared in Fig 4. Furthermore, we found that OOP expenditures alone also pushed patients below the poverty line (Fig 4): lymphadenitis (17%), pleuritis (13%), meningitis (33%) and others (33%). Overall, the highest proportion of patients falling below the poverty line was among patients with lymphadenitis, followed by those with pleuritis, meningitis, and others (Table E in S1 Text). Using total costs or OOP expenditures, we found no significant difference in disease manifestations associated with falling below the poverty line (p > 0.05). We also noted that patients in higher salary groups were pushed below the poverty line due to EPTB-related total costs or out-of-pocket expenditures (p < 0.001, Table E in S1 Text). The lower-salary groups were below the poverty line even before incurring EPTB-related total spending. While patients from the higher-salaried groups were also pushed below the poverty line (p < 0.001): low-middle (33%), high-middle (19%), high (6%), and very high (3%). Similarly, EPTB-related OOP expenditures also resulted in moving patients below the poverty line: low-middle (27%), high-middle (5%), high (3%) and very high (1%), as shown in Fig 4.

## Discussion

This study pioneered the estimation of EPTB-related CHE categorised by disease manifestation, asset-index-based inequality in EPTB-related costs, and their impact on patients relative to the population-level poverty line. We found that EPTB patients of all manifestations bear significant financial burdens during the pre-diagnostic phase of illness. Our sensitivity analyses using various thresholds also indicated that patients with all EPTB manifestations were at risk of CHE. This finding of higher costs among EPTB patients is similar to those reported in the Netherlands [26]. The higher medication and indirect costs are also comparable to the ones noted in Yemen [21]. Similarly, higher diagnostic costs for EPTB patients are reported in high-, upper-middle- and low-middle-income settings in the Netherlands, Thailand, and Kenya, respectively [14,26,48]. However, a more exhaustive comparison cannot be made, as no literature exists on CHE categorised by EPTB manifestations.

A significant difference categorised by the EPTB manifestations was also evident in the contributors to the total costs. For example, in terms of total costs, patients with lymphadenitis incurred a higher proportion of indirect costs than other patients. Meanwhile, pleuritis, meningitis, and other patients faced more direct (OOP) costs. The EPTB-related OOP expenditures in our study (48–84% among several disease manifestations) are even higher than the reported 63% OOP among the general population in India [49]. This variation may be rooted in more extended treatment for meningitis, patients’ health-seeking behaviour and affordability. We found that more severe forms of EPTB manifestations, such as meningitis and pleuritis, may lead to earlier healthcare access. This may prevent EPTB from impacting patients’ working capacity and incur higher indirect costs. Moreover, most of the patients from the lower salary groups had lymphadenitis and may have a less severe illness. We did not have detailed information about the previous diagnostic tests the patient may have undergone. EPTB patients may have delayed treatment due to the cost of fine-needle aspiration cytology required to diagnose lymphadenitis. Based on our available data, it is plausible that, because their disease was less severe, these patients may have continued to delay accessing healthcare until the disease affected their work capacity. These findings are similar to those reported among EPTB patients in Zanzibar [13].

We also found that factors related to the individual patient, their healthcare access and utilisation were associated with the CHE. For example, longer duration and a higher percentage reduction in working capacity were associated with the CHE. Similarly, we found an association between the lower salary groups and the CHE among EPTB patients. As in our study, a higher proportion of EPTB patients with reduced working capacity than with lymphadenitis was reported in Zanzibar [13]. These factors (working capacity and salary) may be closely related to the CHE. For example, these low-salaried lymphadenitis patients may not be able to afford an efficient healthcare pathway that could lead to an earlier diagnosis. Thus, due to affordability issues, patients continue to delay accessing healthcare until their working capacity is significantly affected. Such a delay could also lead to higher indirect costs, as we found in our study. Otherwise, if patients accessed relatively better-resourced HFs, even the regional or private hospitals deemed more expensive, we found such HFs associated with lower CHE. Hence, if patients could afford and were able to access relatively well-equipped HFs, an earlier diagnosis may lessen the chances of CHE.

Our inequality analysis revealed a similar difference in cumulative costs. Based on the asset index, we found that pleuritis and meningitis patients with higher SEPs accounted for a larger proportion of several cost contributors. Meanwhile, patients with lymphadenitis incurred higher cumulative costs in the lower SEPs. These findings are striking, as they show variation in EPTB-related expenses across manifestations and differences in the contributors to these costs among patients with multiple SEPs. Thus, an EPTB diagnosis and treatment appear to be linked to patients’ SEPs and affordability rather than to free-of-cost availability, per the WHO’s end-TB strategy.

Our analysis found that patients of all disease manifestations and salary groups were vulnerable to impoverishment and could fall into the illness-related poverty trap. We noted a higher proportion of lymphadenitis (62–68%) and a higher proportion of low-salaried (20–48%) EPTB patients pushed below the poverty line due to EPTB-related costs than those reported in India (22%) [46,49]. We did not record subsequent salaries and could not ascertain if the lowest salary group fell into extreme poverty; however, it cannot be ruled out. Moreover, a longitudinal record of income following the illness could have informed the time taken to recover from the poverty trap.

Contributors to EPTB-related indirect costs have not been reported in the past. It is important to note that the implications of illness extended beyond the patients. Instead, they also affected their family members, who served as patients’ caregivers. Overall, 28% of the indirect costs were due to the loss of working capacity among family members. We found the highest proportion of indirect costs (36%) due to the forgone productivity of family members of meningitis patients. This may indicate that a more severe illness, such as meningitis, increase patients’ dependence on caregivers. Finally, it is essential to note that the higher direct (out-of-pocket) and indirect costs were not limited to the EPTB patients. The non-TB presumptive patients also faced high costs. While EPTB patients may receive treatment free of cost, the hardships of non-TB patients to get their diagnosis and treatment may continue. Thus, a wide variation in EPTB signs and symptoms may mimic other illnesses, further complicating patients’ ability to receive appropriate diagnoses.

Meanwhile, as we found in our study cohort, some groups may be disproportionately more affected during this pre-diagnostic phase of illness. This included traditionally less-empowered women, worse-off patients with low education levels and economically active young adults. All these factors may compound into household-level financial implications.

Our study also has some limitations. We did not have information on coping mechanisms to address the costs patients incur. Such mechanisms may affect patients’ ability to access healthcare. However, coping mechanisms are not known to play a significant role in chronic illnesses such as EPTB. Coping mechanisms that may reflect resilience towards EPTB-related expenditures were not available. The CHE and a substantial proportion of patients falling below the poverty line (per several thresholds) are alarming. Such an impact pushes patients into the poverty trap. Patients from the lower-income group are often supported in the CHE. However, we found that the catastrophic effects of EPTB span across other income groups as well. Our data on annual income were self-reported by patients and may not be precisely accurate due to informal sources of income in rural areas. Therefore, we also estimated and utilised the asset index for an inequality analysis, which yielded results consistent with significant cost variation across EPTB manifestations. We recorded individual patients’ income rather than household income. This could affect the estimation of CHE. However, we conducted sensitivity analyses to address this issue, and our findings remained unchanged. Due to the general unavailability of EPTB-related cost estimates, we could not compare costs for patients in this geographical setting. Our study site mainly served rural areas. Such areas may have a higher proportion of patients with poor income and low education levels, which is peculiar to TB, known as the disease of poverty. However, our findings may be generalised with caution in urban settings.

Another limitation was the lack of information about the contributors to the cost, even for the PTB, in our study settings. Moreover, we did not have a detailed breakdown of the diagnostic costs. This prevented drawing any comparisons, even between the expenses incurred by PTB patients and those incurred by EPTB patients.

Estimating the direct (OOP) and indirect cost contributors to EPTB is a major strength of this study. An analysis of OOP costs, forgone working capacity and its financial implications for EPTB has yet to be reported in such depth. This study provides policymakers with evidence comparable to that for other illnesses that may also have a chronic course and affect patients’ and caregivers’ productivity. The disaggregation of costs would support the development of appropriate policies and address the demand side of financial barriers, thereby improving timely access to healthcare. A lack of financial protection due to the CHE is a significant impediment to achieving end-TB goals. Unless EPTB gains a focus like the PTB, patient-incurred costs will continue to be detrimental to household finances, especially for patients who are already worse off. Moreover, ensuring that EPTB signs and symptoms are taken into account and providing timely diagnostics to all presumptive patients can only be achieved if universal health coverage (UHC) is improved and expanded.

## Conclusion

This study provides comprehensive evidence of the substantial and unequal financial burden borne by EPTB patients in India, which varies significantly by disease manifestation and socioeconomic status. Patients with pleuritis, meningitis, and other severe forms of EPTB incurred markedly higher OOP and indirect costs compared to those with lymphadenitis. The majority of these patients experienced CHE, and a considerable proportion were pushed below the poverty line, highlighting a profound vulnerability to impoverishment even among relatively higher-income groups. We also found that presumptive non-TB patients incurred high pre-diagnostic costs, despite ultimately not receiving a TB diagnosis. This finding underscores a gap in financial protection for individuals with chronic, TB-like symptoms who remain outside the support of TB programs. Overall, our findings demonstrate that EPTB and non-TB diagnostic pathways are financially burdensome, especially for lower socioeconomic groups. We conclude that addressing these challenges requires integrated health financing strategies that extend beyond TB treatment, including improved access to diagnostics, reduced patient costs, and progress toward universal health coverage. Such efforts are essential not only for equity in TB care but also for alleviating broader healthcare-related financial hardship among underserved populations.

## Supporting information

S1 Data Dataset. (CSV)

S1 Text Supplementary data analysis. (PDF)
