# Supplementary material for: The catastrophic financial burden of extrapulmonary tuberculosis and asset index-based inequality analysis: A prospective cohort study analysing patients’ impoverishment
Source: PLOS Glob Public Health. 2026 Feb 9;6(2):e0005730. doi: 10.1371/journal.pgph.0005730 (PMC12885288; doi:10.1371/journal.pgph.0005730)
Supplement: S1 Text — (PDF) [file pgph.0005730.s002.pdf]

Table A: Median (IQR) time taken (hours) for the diagnostic visit

	Lymphadenitis	Pleuritis	Meningitis	Others	Total	p-value*
**EPTB patients**	6.5 (4.0 to 10.0)	72.0 (24.0 to 96.0)	84.0 (48.0 to 102.0)	108.0 (54.0 to 144.0)	8.0 (5.0 to 48.0)	<0.001
**Non-TB patients**	6.0 (4.0 to 10.0)	84.0 (48.0 to 120.0)	72.0 (72.0 to 96.0)	NA	7.8 (4.0 to 12.8)	<0.001

* p-value corresponds to Kruskal Wallis test

Table B: Ingredient of total direct & Indirect costs (USD) of EPTB manifestations

		Lymphadenitis	Pleuritis	Meningitis	Others	p-value*
Admission HF	Median (IQR)	0.0 (0.0 to 0.0)	0.0 (0.0 to 23.1)	0.0 (0.0 to 25.7)	37.0 (20.6 to 48.1)	<0.001
Consultation	Median (IQR)	2.1 (0.0 to 5.1)	7.2 (1.0 to 10.3)	6.2 (4.8 to 10.3)	8.7 (3.3 to 14.1)	<0.001
Medicines	Median (IQR)	5.1 (3.1 to 12.3)	15.4 (3.1 to 31.9)	11.3 (5.9 to 30.9)	43.7 (41.1 to 50.1)	<0.001
Diagnostics	Median (IQR)	2.8 (1.1 to 7.2)	9.3 (0.8 to 15.9)	10.3 (3.1 to 24.2)	25.7 (15.2 to 38.6)	<0.001
Transportation	Median (IQR)	1.0 (0.5 to 3.1)	2.6 (1.0 to 5.1)	2.6 (2.1 to 4.9)	10.3 (6.4 to 10.3)	<0.001
Transportation follow-up visits	Median (IQR)	4.0 (2.1 to 6.4)	53.5 (12.3 to 77.1)	59.9 (39.4 to 85.5)	76.3 (43.4 to 110.5)	<0.001
Total direct costs (Out of pocket)	Median (IQR)	18.7 (8.4 to 37.4)	86.1 (61.1 to 146.3)	117.8 (82.1 to 145.5)	241.1 (159.1 to 299.8)	<0.001
Travel and waiting time for the diagnostic visit	Median (IQR)	0.2 (0.1 to 0.3)	2.6 (0.6 to 3.8)	1.8 (1.2 to 2.5)	3.7 (2.1 to 5.4)	<0.001
Productivity lost by EPTB patients	Median (IQR)	12.9 (7.0 to 28.5)	23.0 (13.6 to 35.1)	19.3 (14.5 to 33.9)	25.0 (18.1 to 38.8)	0.015
Productivity lost by EPTB patients' Family Member	Median (IQR)	5.3 (1.2 to 13.1)	8.8 (5.1 to 17.5)	13.6 (9.1 to 15.9)	6.0 (1.6 to 10.0)	0.016
Total Indirect costs	Median (IQR)	21.2 (11.9 to 37.1)	34.2 (22.7 to 53.5)	34.8 (25.8 to 58.8)	37.6 (21.7 to 55.7)	0.003

* p-value corresponds to Kruskal Wallis test

Table C: Proportion of various ingredients of the total direct & Indirect costs by EPTB manifestations

				Lymphadenitis n = 142 % (SD)	Pleuritis n = 40 % (SD)	Meningitis n = 12 % (SD)	Others n = 6 % (SD)	Total n = 200 % (SD)	p-value*
**EPTB patients**	**Direct costs** (OOP expenditures)	Pre-treatment	Admission HF	4 (7.7)	8 (11.1)	7 (8.5)	22 (10.7)	5 (9.2)	<0.001
			Consultation	10 (10.5)	7 (6.4)	7 (8.3)	5 (3.1)	9 (9.6)	0.197
			Medication	30 (17.6)	17 (13.1)	14 (10.3)	26 (12.1)	26 (17.3)	<0.001
			Diagnostics	17 (11.6)	10 (7.7)	11 (9.2)	11 (5.7)	15 (11.1)	0.001
			Transportation	8 (6.4)	4 (3.5)	3 (2.7)	5 (3.8)	7 (6.0)	<0.001
		Post-treatment	Transportation follow-up visits	32 (26.5)	55 (31.2)	57 (27.0)	31 (19.2)	38 (29.1)	<0.001
	**Indirect cost**	Pre-treatment	Diagnostic visit (travel & wait time)	3 (9.8)	7 (6.6)	5 (3.2)	10 (6.3)	4 (9.1)	0.017
			Patients' forgone productivity	69 (21.3)	65 (14.8)	59 (8.6)	78 (11.0)	68 (19.5)	0.172
			Family member (caregiver) lost productivity	29 (21.0)	28 (15.2)	36 (7.8)	12 (7.6)	28 (19.3)	0.115
	**Total cost**		Total direct cost	48 (24.6)	66 (22.8)	73 (12.9)	84 (9.0)	54 (25.3)	<0.001
			Total indirect cost	52 (24.6)	34 (22.8)	27 (12.9)	16 (9.0)	46 (25.3)	<0.001

* p-value corresponds to chi-square test

Table D: Multivariable regression analysis estimating factors associated with the base-case catastrophic expenditures (CHE)

		**Patients facing CHE (%)**	**Patients not facing CHE (%)**	**OR (univariable)**	**OR (multivariable)**
Gender	Female	42 (35.9)	75 (64.1)	-	-
	Male	41 (52.6)	37 (47.4)	0.51 (0.28-0.90, p=0.022)	2.02 (0.45-10.50, p=0.374)
Age groups	Under 15 yr	3 (20.0)	12 (80.0)	-	-
	16-29 yr	33 (42.9)	44 (57.1)	0.33 (0.07-1.15, p=0.109)	0.05 (0.00-0.89, p=0.050)
	30-44	21 (60.0)	14 (40.0)	0.17 (0.03-0.64, p=0.014)	0.08 (0.00-1.52, p=0.102)
	>45	14 (53.8)	12 (46.2)	0.21 (0.04-0.86, p=0.042)	0.08 (0.00-1.80, p=0.130)
Education level	Primary or below	56 (47.1)	63 (52.9)	-	-
	Middle or Secondary	21 (38.9)	33 (61.1)	1.40 (0.73-2.72, p=0.317)	1.01 (0.23-4.23, p=0.993)
	Higher	6 (27.3)	16 (72.7)	2.37 (0.91-6.99, p=0.092)	0.33 (0.05-2.13, p=0.243)
Marital status	Married	57 (48.3)	61 (51.7)	-	-
	Unmarried	22 (37.9)	36 (62.1)	1.53 (0.81-2.93, p=0.195)	4.84 (0.83-33.12, p=0.089)
Household family members	1-4	30 (46.2)	35 (53.8)	-	-
	5-7	36 (37.9)	59 (62.1)	1.40 (0.74-2.67, p=0.298)	1.34 (0.35-5.22, p=0.667)
	>=8	17 (48.6)	18 (51.4)	0.91 (0.40-2.08, p=0.817)	1.56 (0.29-8.85, p=0.602)
Salary category	High	24 (36.9)	41 (63.1)	-	-
	Very high	19 (35.8)	34 (64.2)	1.05 (0.49-2.24, p=0.904)	0.60 (0.14-2.56, p=0.494)
	High-middle	19 (52.8)	17 (47.2)	0.52 (0.23-1.19, p=0.125)	0.35 (0.06-1.79, p=0.221)
	Low-middle, Low & Very low	21 (51.2)	20 (48.8)	0.56 (0.25-1.23, p=0.149)	0.07 (0.01-0.42, p=0.006)
Occupation	Govt Employed	14 (40.0)	21 (60.0)	-	-
	Housewife	21 (32.8)	43 (67.2)	1.37 (0.58-3.21, p=0.475)	9.86 (1.19-98.49, p=0.040)
	Unemployed	18 (41.9)	25 (58.1)	0.93 (0.37-2.30, p=0.868)	1.84 (0.24-14.28, p=0.553)
	Private Employed	30 (56.6)	23 (43.4)	0.51 (0.21-1.21, p=0.129)	2.15 (0.37-13.22, p=0.394)
Patient history of TB	No	72 (42.1)	99 (57.9)	-	-
	Yes	11 (45.8)	13 (54.2)	0.86 (0.36-2.06, p=0.730)	33.23 (1.75-919.30, p=0.026)
	No	65 (40.9)	94 (59.1)	-	-

Family member's history of TB	Yes	18 (50.0)	18 (50.0)	0.69 (0.33-1.43, p=0.319)	0.81 (0.17-3.96, p=0.794)
Self-medication	No	62 (39.7)	94 (60.3)	-	-
	Yes	21 (53.8)	18 (46.2)	0.57 (0.28-1.14, p=0.114)	0.64 (0.16-2.51, p=0.518)
Hospitalisation status	Inpatient	54 (71.1)	22 (28.9)	-	-
	Outpatient	29 (24.4)	90 (75.6)	7.62 (4.04-14.84, p<0.001)	6.92 (1.73-31.83, p=0.008)
Co-morbidities (DM, HIV, previous TB)	No	61 (38.9)	96 (61.1)	-	-
	Yes	22 (57.9)	16 (42.1)	0.46 (0.22-0.94, p=0.035)	0.02 (0.00-0.32, p=0.008)
Patient level delay	Above Median PD	35 (38.0)	57 (62.0)	-	-
	Below Median PD	48 (46.6)	55 (53.4)	0.70 (0.40-1.24, p=0.228)	1.51 (0.43-5.50, p=0.520)
Health system level-I delay	Above Median SD	48 (47.1)	54 (52.9)	-	-
	Below Median SD	35 (37.6)	58 (62.4)	1.47 (0.83-2.62, p=0.184)	1.14 (0.26-5.20, p=0.862)
Health system level-II delay	Below Median SD	46 (40.0)	69 (60.0)	-	-
	Above Median SD	37 (46.2)	43 (53.8)	0.77 (0.43-1.38, p=0.386)	1.43 (0.39-5.45, p=0.586)
Number of HFs previously visited for this illness	One	26 (38.8)	41 (61.2)	-	-
	Two-three	42 (53.8)	36 (46.2)	0.54 (0.28-1.05, p=0.072)	0.39 (0.04-3.18, p=0.385)
	Missing	15 (30.0)	35 (70.0)	1.48 (0.68-3.27, p=0.324)	1.62 (0.07-29.31, p=0.749)
Number of previous visits to HFs for this illness	One	23 (39.7)	35 (60.3)	-	-
	Two-Four	48 (50.5)	47 (49.5)	0.64 (0.33-1.24, p=0.192)	1.62 (0.20-14.41, p=0.653)
	Missing	12 (28.6)	30 (71.4)	1.64 (0.71-3.93, p=0.253)	1.14 (0.07-17.66, p=0.927)
Time to reach the nearest HF	Below 30 min	26 (35.1)	48 (64.9)	-	-
	Between 30- 60 min	22 (30.6)	50 (69.4)	1.23 (0.62-2.47, p=0.556)	3.35 (0.84-16.53, p=0.106)
	Above 60 min	35 (71.4)	14 (28.6)	0.22 (0.10-0.47, p<0.001)	0.12 (0.02-0.63, p=0.016)
Time to reach the diagnostic site	Below 30 min	33 (34.7)	62 (65.3)	-	-
	30- 60 min	19 (34.5)	36 (65.5)	1.01 (0.50-2.05, p=0.981)	3.31 (0.63-20.74, p=0.173)
	Above 60 min	31 (68.9)	14 (31.1)	0.24 (0.11-0.51, p<0.001)	1.51 (0.29-8.50, p=0.627)

Total time (travel & wait) at the diagnostic site	Below 60 min	24 (21.1)	90 (78.9)	-	-
	Above 60 min	59 (72.8)	22 (27.2)	0.10 (0.05-0.19, p<0.001)	0.17 (0.04-0.72, p=0.019)
HFs previously visited for this illness	Dispensary	48 (49.5)	49 (50.5)	-	-
	Health Center/District/Others	6 (28.6)	15 (71.4)	2.45 (0.91-7.35, p=0.087)	3.63 (0.48-33.01, p=0.227)
	Regional/Private Hospital	29 (37.7)	48 (62.3)	1.62 (0.89-3.00, p=0.120)	1.34 (0.26-7.09, p=0.722)
Reduction is working capacity duration (days)	Below 15 days	28 (30.8)	63 (69.2)	-	-
	Above 15 days	55 (52.9)	49 (47.1)	0.40 (0.22-0.71, p=0.002)	0.09 (0.02-0.36, p=0.002)
Reduction is working capacity extent (%)	Below 25%	2 (9.5)	19 (90.5)	-	-
	25- 50%	26 (27.7)	68 (72.3)	0.28 (0.04-1.04, p=0.097)	0.01 (0.00-0.47, p=0.025)
	Above 50%	55 (68.8)	25 (31.2)	0.05 (0.01-0.18, p<0.001)	0.00 (0.00-0.09, p=0.003)

Table E: EPTB patients pushed below the poverty line due to EPTB-related costs

			Patients above poverty line (%)	Patients below poverty line (%)	*p-value
**Total costs**	EPTB manifestations	Lymphadenitis	111 (73.0)	27 (61.4)	0.288
		Pleuritis	30 (19.7)	10 (22.7)	
		Meningitis	7 (4.6)	5 (11.4)	
		Others	4 (2.6)	2 (4.5)	
**Out-of-pocket**	EPTB manifestations	Lymphadenitis	118 (71.5)	24 (68.6)	0.278
		Pleuritis	35 (21.2)	5 (14.3)	
		Meningitis	8 (4.8)	4 (11.4)	
		Others	4 (2.4)	2 (5.7)	
**Total costs**	Salary categories	Very low	0 (0.0)	9 (20.5)	<0.001
		Low	0 (0.0)	17 (38.6)	
		Low-middle	10 (6.6)	5 (11.4)	
		High-middle	29 (19.1)	7 (15.9)	
		High	62 (40.8)	4 (9.1)	
		Very high	51 (33.6)	2 (4.5)	
**Out-of-pocket**	Salary categories	Very low	0 (0.0)	9 (25.7)	<0.001
		Low	0 (0.0)	17 (48.6)	
		Low-middle	11 (6.7)	4 (11.4)	
		High-middle	35 (21.2)	2 (5.7)	
		High	67 (40.6)	2 (5.7)	
		Very high	52 (31.5)	1 (2.9)	

* p-value corresponds to chi-square test
